# L'hémangiome caverneux costal: une tumeur rare de la paroi thoracique

**Published:** 2012-04-22

**Authors:** Mohammed Bouchikh, Abdellah Achir, Khadija Setti, Tchely-Oualy Mbola, Damsane Lamboni, Fouad Zouaidia, Najat Mahassini, Abdellatif Benosman

**Affiliations:** 1 Service de Chirurgie Thoracique, CHU Ibn Sina, Rabat, Maroc; 2 Laboratoire Central d'Anatomie et de Cytopathologie, CHU Ibn Sina, Rabat, Maroc

**Keywords:** Paroi thoracique, Tumeur costale, Hémangiome, Hamartome, Maroc

## Abstract

L′hémangiome caverneux de l′os est une tumeur bénigne rare; sa localisation costale est exceptionnelle. Nous rapportons le cas d′un patient de 17 ans qui consultait pour une masse pariétale. La tomodensitométrie a objectivé une tumeur de la 6^e^ côte droite avec une rupture partielle de la corticale osseuse. Le traitement a consisté en une résection chirurgicale et l′examen microscopique de la pièce opératoire a conclu à un hémangiome costal de type caverneux. Aucune récidive n′a été notée 7 mois après l′intervention. L′hémangiome costal est une tumeur rare de la paroi thoracique. Son diagnostic de certitude est histopathologique car l'imagerie peut prêter confusion avec une origine maligne ou infectieuse.

## Introduction

L′hémangiome osseux est un hamartome vasculaire bénin représentant moins de 1% de l′ensemble des tumeurs osseuses; sa localisation costale est extrêmement rare [1–2]. Nous rapportons un cas d′hémangiome costal et nous en discuterons les aspects cliniques, radiologiques et thérapeutiques.

## Patient observation

**Figure 1:**
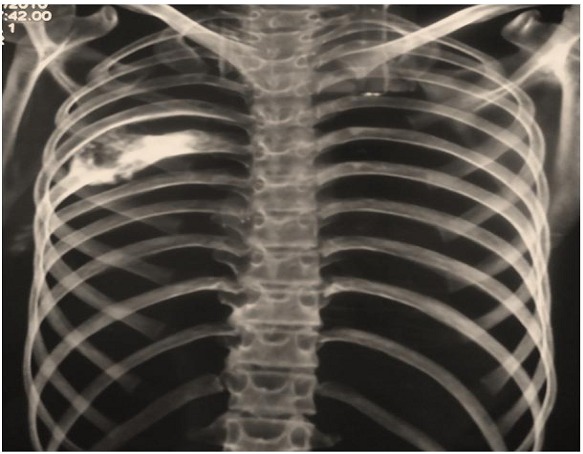
Reconstruction tridimensionnelle de la cage thoracique montrant une tumeur au dépend de l'arc postérieur de la 6e côte droite

Il s′agissait d′un patient de 17 ans qui rapportait l′apparition 2 ans avant sa consultation; d′une tuméfaction dorsale augmentant progressivement de volume. Cette masse était indolore, fixe par rapport au plan profond et mesurait environ 10cm de diamètre. La tomodensitométrie thoracique objectivait une tumeur expansive et lytique avec une corticale partiellement interrompue, au dépend de l′arc postérieur de la 6ème côte droite et arrivant jusqu′au contact de l′articulation costo-vertebrale (Figure 1).

Comme la tumeur était bien circonscrite une intervention chirurgicale à visée diagnostique et thérapeutique était décidée. On avait réalisé alors une résection en mononbloc des arcs postérieurs des 5e, 6e et 7e côtes droites et de l′apophyse transverse de la 6e vertèbre passant à distance de la tumeur. Le defect pariétal a été réparé par une prothèse de Mercilène^®^ recouverte par un lambeau du muscle grand dorsal.

**Figure 2:**
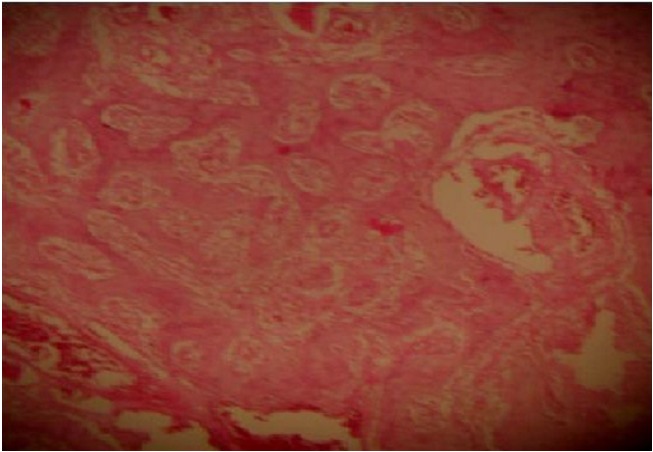
Prolifération tumorale faite de nombreux vaisseaux entremêlés au sein de travées osseuses régulières et normocalcifiées (Coloration à l'hématoxyline éosine Grossissement×10)

L′examen histopathologique révélait un tissu osseux fait de travées régulières et normocalcifiées entre lesquelles siège une prolifération tumorale, faite de vaisseaux de grande taille gorgés de sang et bordés de cellules endothéliales régulières. Le stroma était grêle ponctué de quelques éléments inflammatoires mononuclés (Figure 2). Cet aspect morphologique était celui d′un hémangiome caverneux interosseux.

Les suites opératoires étaient simples et aucune récidive n′a été notée après un recul de 7 mois.

## Discussion

L′hémangiome costal est une tumeur rare représentant moins de 1% de l′ensemble des tumeurs costales primitives [2]. On en décrit deux types anatomopathologiques: le type caverneux comme celui rapporté dans notre cas et le type capillaire, plus rare, constitué par un ensemble de petits vaisseaux tortueux bordés par un endothélium unistratifié [3].

Les hémangiomes costaux sont généralement asymptomatiques découverts fortuitement sur une radiographie thoracique [1–3]. Ils peuvent être diagnostiqués après l′apparition d′une masse pariétale comme chez notre patient, ou après des signes de compression nerveuse ou vasculaire [4, 5].

La radiographie thoracique permet de poser le diagnostic d′une tumeur costale sous forme d′une lésion expansive, lytique soufflant la corticale. A la tomodensitométrie thoracique l′hémangiome apparaît comme une tumeur expansive de densité hétérogène avec des fines travées osseuses donnant un aspect en "nid d′abeilles" ou en "rayon de miel" [1]. Des cas de destruction de la corticale, comme le nôtre, ont été rapportés dans la littérature ce qui peut faire penser à un processus malin [6]. A l′imagerie par résonance magnétique cette tumeur donne un hyposignal en T1, un hypersignal en T2 et se rehausse fortement après injection de gadolinium [1].

Les images radiologiques ne sont pas spécifiques et peuvent poser le problème de diagnostic différentiel avec des tumeurs malignes comme l′ostéosarcome ou le sarcome d′Ewing, des tumeurs bénignes comme le kyste anévrysmal, la dysplasie fibreuse ou avec des processus infectieux et parasitaires comme l′hydatidose ou la tuberculose costale [1, 3, 4, 7].

Le diagnostic définitif est apporté par l′étude anatomopathologique d′une biopsie ou d′une pièce d′exérèse. Le traitement des hémangiomes costaux est chirurgical. Il consiste en exérèse complète de la tumeur pour éviter une éventuelle récidive. Pour les tumeurs d′accès difficile comme celles de la première côte, d′autres alternatives sont proposées à savoir la radiothérapie, l′embolisation artérielle et l′injection d′alcool [1].

Le pronostic de ces tumeurs est généralement bon [1–6]. Aucun cas de transformation maligne n′a été rapporté dans la littérature.

## Conclusion

L'hémangiome costal est une tumeur très rare. Son diagnostic est histopathologique car l′imagerie peut prêter confusion avec une origine maligne ou infectieuse. Son pronostic est bon après une exérèse chirurgicale complète.
